# Visualising the expansion and spread of coronavirus disease 2019 by cartograms

**DOI:** 10.1177/0308518X20910162

**Published:** 2020-02-26

**Authors:** Peichao Gao, Hong Zhang, Zhiwei Wu, Jicheng Wang

**Affiliations:** State Key Laboratory of Earth Surface Processes and Resource Ecology, Beijing Normal University, China; Faculty of Geographical Science, Beijing Normal University, PR China; Faculty of Geosciences and Environmental Engineering, Southwest Jiaotong University, PR China; Faculty of Geosciences and Environmental Engineering, Southwest Jiaotong University, PR China; Department of Land Surveying and Geo-Informatics, The Hong Kong Polytechnic University, Hong Kong; Faculty of Geosciences and Environmental Engineering, Southwest Jiaotong University, PR China

**Keywords:** Cartogram, visualisation, graphic

## Abstract

Coronavirus disease 2019 (COVID-19) has emerged as a growing focus of global attention and a critical factor in public-health decision making. Towards fighting the COVID-19 outbreak, countries worldwide and international organisations have taken various actions, including promoting the transparency of and public access to disease data. In such public communications, maps have played an important role in that a map is worth a thousand words. Most of these have taken the form of a choropleth map. Here, we propose employing cartograms to visualise both the expansion and spread of COVID-19. We designed a combination of six circular cartograms containing the data of confirmed cases every 48 hours from 24 January to 3 February 2020. Such a design conveys both spatial and temporal information more intuitively and efficiently, so it can be expected to facilitate better public participation in the fight against COVID-19.

Fighting the coronavirus disease 2019 (COVID-19) outbreak has very recently emerged as a critical challenge for China and the focus of global attention. Named by the World Health Organization (WHO, 2020), COVID-19 (whose earlier temporary name was 2019-nCoV) is a novel coronavirus identified with close genetic relatedness to severe acute respiratory syndrome and is highly contagious. By 7 February 2020, more than 30,000 confirmed cases of COVID-19 had been reported for globally, according to the Situation Report of the WHO. Most of these confirmed cases are from China, especially the Hubei province. In the fight against COVID-19, one important strategy is to keep the public informed on progress via the media, including maps and graphics.

Here, we propose visualising the expansion and spread of COVID-19 using area cartograms. The study area is 31 Chinese provinces (or equivalent administrative units), excluding Hong Kong, Macaw and Taiwan. These 31 provinces were selected because of the same data source, namely provincial health commissions. In employing an area cartogram, we identified three major types: contiguous, non-contiguous and circular (also called the Dorling cartogram; Dorling, 1996; Gao et al., 2019). We tested all three types, finding that the most appropriate in our case is the circular cartogram. To make it easier to distinguish between different circles, we coloured circular cartograms using a scientific scheme designed by Harrower and Brewer (2003), with two constraints: (a) adjacent circles do not share the same colour, and (b) the colour of each province remains stable among different cartograms.

The result is shown in Figure 1, which is a combination of six circular cartograms. These cartograms were created with data from 24 January, 26 January, 28 January, 30 January 1 February, and 3 February 2020 (24:00, UTC +8), respectively. In each cartogram, a circle denotes a province, and its size is proportional to the number of confirmed COVID-19 cases reported from that province. All cartograms were created at the same scale, so it is meaningful to compare the sizes of two circles of different cartograms. Some circles were drawn with the numbers of confirmed cases on them. The two letters drawn on or around each circle denote the standard abbreviation of the corresponding province. Such abbreviations can be found at, for example, https://www.cottongen.org/data/nomenclatures/China_provinces.

**Figure 1.**
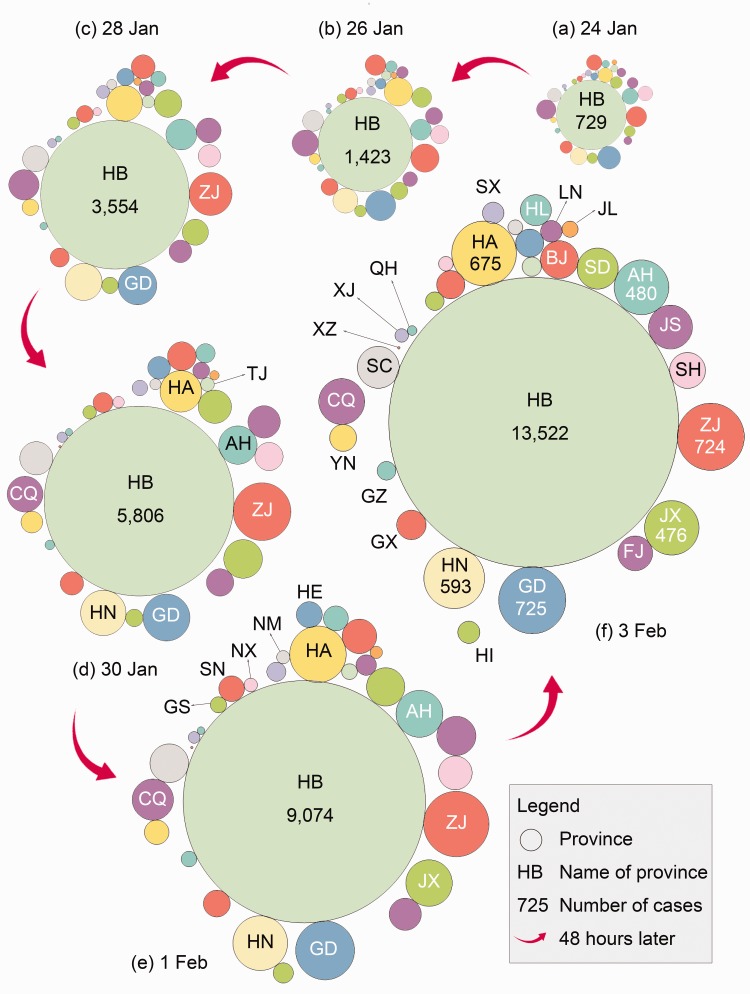
Expansion and spread of coronavirus disease 2019 (COVID-19) in 31 Chinese provinces (data for Hong Kong, Macaw and Taiwan are not visualised) for the period 24 January to 3 February 2020. The numbers of confirmed COVID-19 cases of 31 provinces on (a) 24 January, (b) 26 January, (c) 28 January, (d) 30 January, (e) 1 February and (f) 3 February.

Many observations can be made using Figure 1. For example, the total area of (i.e. space occupied by) Figure 1(c) is much greater than that of Figure 1(b), revealing a considerable increase in the number of confirmed cases over the 48 hours from 26 to 28 January. The circle of Hubei (HB) province has become notably larger, while the expansion of the other circles seems to be less notable. This fact, associated with the media’s news, suggests that COVID-19 probably originated in HB and is spreading to other provinces.
